# Supplementary material for: The interactome of intact mitochondria by cross-linking mass spectrometry provides evidence for coexisting respiratory supercomplexes
Source: Mol Cell Proteomics. 2017 Dec 8;17(2):216–32. doi: 10.1074/mcp.RA117.000470 (PMC5795388; doi:10.1074/mcp.RA117.000470)
Supplement: Supplemental Data [file supp_RA117.000470_133922_0_supp_23978_fzffwf.zip › spectra_annotation/mito_spectra_annotation/396-1-17-1-10-1.pdf]

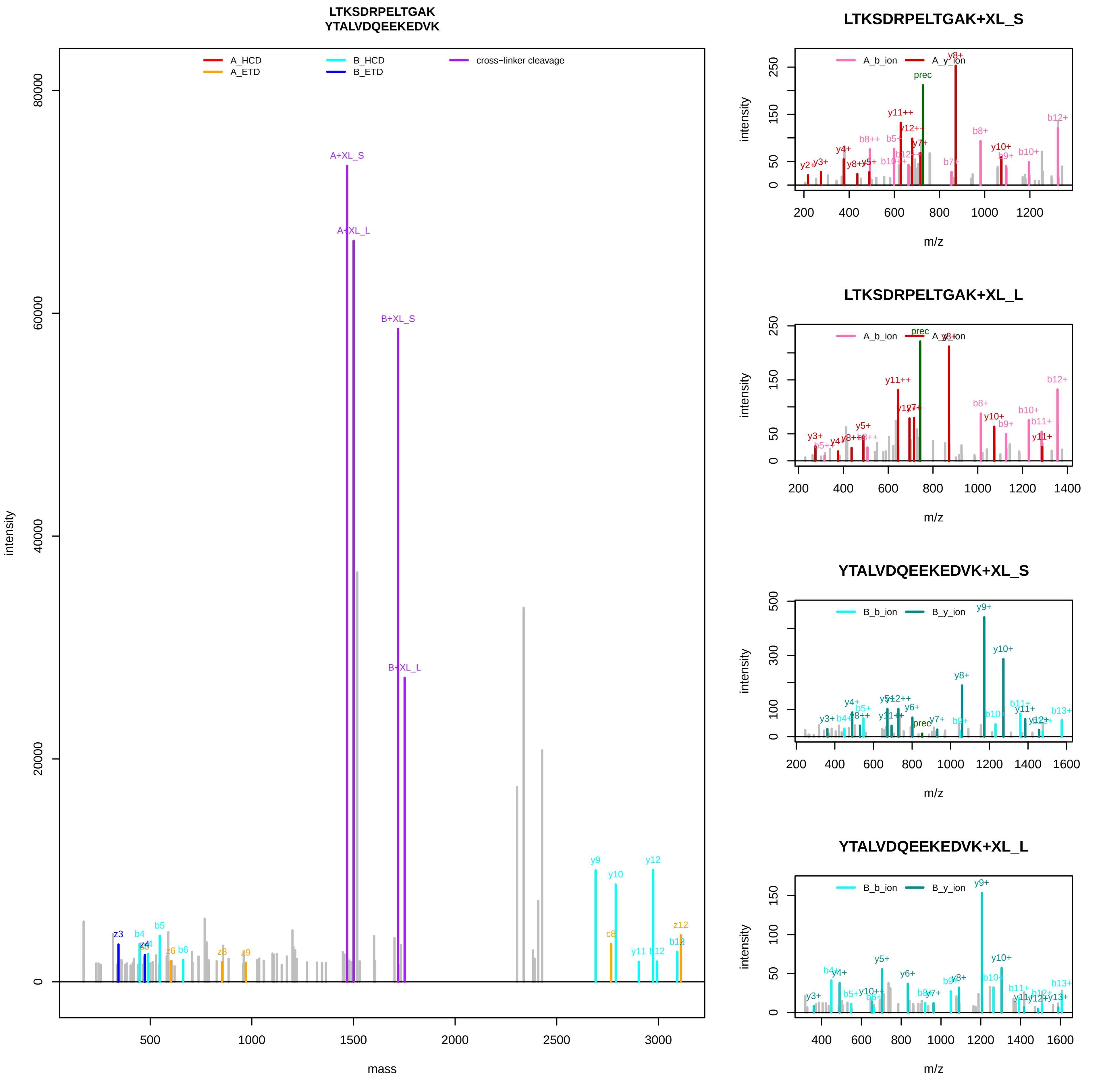
LTKSDRPELTGAK
YTALVDQEEKEDVK
A_HCD
A_ETD
B_HCD
B_ETD
cross−linker cleavage
A+XL_S
A+XL_L
B+XL_S
B+XL_L
z3
b4
z4
b5
z6
b6
z8
z9
y9
y10
c8
y11
y12
b12
b13
z12
intensity
mass
0
20000
40000
60000
80000
500
1000
1500
2000
2500
3000
LTKSDRPELTGAK+XL_S
A_b_ion
A_y_ion
prec
y2+
y3+
y4+
y8+
y5+
b8++
b5+
b10++
y11++
y12++
y7+
y8+
b7+
b8+
y10+
b9+
b10+
b12+
intensity
m/z
LTKSDRPELTGAK+XL_L
A_b_ion
A_y_ion
prec
y3+
b5++
y4+
y8++
y5+
y11++
y8+
b8+
y10+
b9+
b10+
b11+
y11+
b12+
intensity
m/z
YTALVDQEEKEDVK+XL_S
B_b_ion
B_y_ion
y3+
b4+
y4+
b5+
y6+
y7+
y8+
y9+
y10+
b9+
b10+
b11+
y11+
b13+
prec
intensity
m/z
YTALVDQEEKEDVK+XL_L
B_b_ion
B_y_ion
y3+
b4+
y4+
b5+
y5+
y6+
y7+
y8+
y9+
y10+
b10+
b11+
b12+
y13+
b13+
intensity
m/z
